# Chemo-Diversity Landscape Using Physico-Biochemical, Elemental, and Metabolic Profiling in Different Stages and Accessions of *Madhuca longifolia* Flowers for Unveiling Their Processing Value and Utilization

**DOI:** 10.3390/molecules31111977

**Published:** 2026-06-05

**Authors:** Shalini Purwar, Ankit Verma, Ravi Prakash Jaiswal, Vigya Mishra, Vishal Chugh, Chandra Mohan Singh, Akbare Azam, Nitin Kumar, Priti Upadhyay, Tribhuvan Chaubey, Ashutosh Rai

**Affiliations:** 1 Department of Basic and Social Science, College of Forestry, BUAT, Banda 210001, Uttar Pradesh, India; purwarshalini@gmail.com (S.P.); ankitvermamadanpur@gmail.com (A.V.); 2 Department of Basic and Social Science, College of Horticulture, BUAT, Banda 210001, Uttar Pradesh, India; vishalchugh3@gmail.com; 3 Department of Chemistry, Government Girl’s P.G. College, Ghazipur 233001, Uttar Pradesh, India; ravi.fare@gmail.com (R.P.J.); akbar_bhu@rediffmail.com (A.A.); 4 Department of Post-Harvest Technology, College of Horticulture, BUAT, Banda 210001, Uttar Pradesh, India; drvigya.hort@gmail.com; 5 Department of Genetics and Plant Breeding, College of Agriculture, BUAT, Banda 210001, Uttar Pradesh, India; cmsingh.gpb@gmail.com (C.M.S.); nitinbawariya1205@gmail.com (N.K.); 6 Division of Vegetable Science, ICAR-Indian Agricultural Research Institute, Pusa, New Delhi 110012, India; priti.iasbhu@gmail.com; 7 ICAR-Indian Institute of Vegetable Research, Varanasi 221305, Uttar Pradesh, India; tribhuvan.chaubey@icar.org.in

**Keywords:** *Madhuca longifolia*, flower, ICPMS, gas chromatography, metabolites, minerals

## Abstract

Variations in sweetness and bitterness among *Madhuca longifolia* flowers strongly influence their processing value and market acceptance, yet the chemo-diversity underlying these traits remains poorly characterized. This study aimed to unravel accession- and stage-specific differences by integrating physico-biochemical, elemental, and metabolic profiling across thirteen accessions (BM-1 to BM-13) from BUAT, Banda. Sensory and textural evaluations revealed wide diversity, with BM-5 displaying superior sweetness and aroma, whereas BM-6, BM-7, and BM-10 were differentiated by firmness, elasticity, and gumminess. Biochemical analyses across flower development showed that BM-5 consistently maintained higher sugars and β-carotene, while BM-1 exhibited marked reductions in sugars and total phenolics content; meanwhile, antioxidant activity increased with maturity, with BM-5 remaining the most stable. ICP-MS elemental analysis confirmed BM-5 as mineral-rich compared with lower-performing accessions. GC-MS metabolomic profiling of contrasting accessions (BM-1 and BM-5) across stages identified 303 volatile and semi-volatile metabolites, and multivariate analyses (PCA, VIP, volcano plots, pathway enrichment) revealed distinct stage- and accession-dependent patterns. Mature BM-5 was enriched in fermentation- and aroma-related metabolites such as melibiose, furfural, 5-HMF, and furaneol, whereas BM-1 accumulated defense-linked compounds including catechol, benzyl nitrile, and maltol. Overall, the integrated chemo-diversity landscape identifies BM-5 as a superior accession with high processing potential and value-addition prospects.

## 1. Introduction

*Madhuca longifolia* J.F. Macbr. (Mahua)plays a significant role in the socioeconomic development and nutritional security of ethnic tribes across several Southeast Asian countries, including India, Indonesia, Myanmar, Sri Lanka, and Nepal [1]. *M. longifolia* flowers are edible and rich in fermentable sugars and phyto-chemicals, making them an excellent food supplement. Traditionally, they are consumed in various forms, including raw, roasted, boiled, and processed [2]. According to the Forest Governance Learning Group India (2018), out of 490,000 tons of *M. longifolia* flowers harvested annually, only 85,000 tons are utilized, and approximately 17.55% of India’s workforce is engaged in *M. longifolia* flower collection [3]. Dried *M. longifolia* flowers represent a low-cost renewable biomass that could serve as a carbon source, although their cost-effectiveness depends on carbohydrate content and recovery efficiency relative to purified glucose [4].

Fermentation plays a crucial role in the food and beverage industries by enhancing sensory appeal, nutritional quality, and shelf life. Consumers increasingly prefer fermented juices due to their improved flavor and enriched vitamin and mineral content [1]. Technological advancements have facilitated the development of value-added products from underutilized biomaterials, with studies reporting effective pre- and post-fermentation strategies that enhance bioactive compounds and functional attributes [1,5,6]. Several investigations have examined the composition and quality of *M. longifolia* flowers, including their antioxidant activity, mineral content, and chemical constituents [4]; their application in producing low-alcoholic beverages [7]; fumaric acid extraction using *Rhizopus oryzae* [4]; and the identification of *Meyerozyma caribbica* M72 for saccharification and ethanol production [8].

*M. longifolia* flowers hold significant cultural and economic importance across tribal and rural India. However, considerable variation exists among different *M. longifolia* accessions in terms of sweetness, bitterness, and the metabolite profiles that shape their aroma, flavor, fermentability, and nutritional properties. These sensory and biochemical differences directly influence both market value and processing suitability, yet they cannot be adequately assessed through conventional sensory tests or basic chemical analyses alone. Therefore, advanced analytical approaches are essential for objective characterization. Gas chromatography (GC/GC–MS) is particularly valuable, as it provides precise qualitative and quantitative profiling of key metabolites, enabling reliable comparison between varieties and supporting quality assurance for diverse industrial applications.

In the present study, we integrate sensory and instrumental texture profiling with biochemical analyses to explore the primary metabolites, mineral composition, and volatile profiles of *M. longifolia* flowers across developmental stages in contrasting accessions. The volatile metabolite profiling was performed using GC–MS, while mineral quantification was achieved through ICP-MS, resulting in a comprehensive dataset for examining developmental dynamics. Variable importance in projection (VIP) analysis was used to identify the most influential GC–MS features contributing to sample variation, enabling the selection of significant biomarkers and enhancing model interpretability in complex chromatographic datasets. To further understand metabolic changes, KEGG pathway mapping was conducted to reveal regulatory networks associated with flower maturation. Through the integration of statistical modeling and metabolic pathway analysis, this study provides a holistic understanding of how primary metabolites, minerals, and volatile compounds are dynamically regulated during floral development, highlighting accession-specific metabolic signatures relevant for conservation, valorization, and potential industrial applications of *M. longifolia*.

## 2. Results

Thirteen accessions (BM-1 to BM-13) of *M. longifolia* collected from the BUAT orchard were evaluated for textural and sensory attributes. Based on these assessments, selected accessions were further subjected to biochemical analyses at two developmental stages, namely immature (IMF) and mature flower (MF) stages. Elemental profiling using ICP-MS was conducted at the mature flower stage. In addition, selected contrasting accessions were subjected to GC–MS analysis at both developmental stages to elucidate the metabolic basis of sensory and aroma attributes.

### 2.1. Texture and Sensory Analysis of Flowers from Different Accessions of M. longifolia

Texture analysis of *M. longifolia* flower accessions (BM-1 to BM-13) collected from the BUAT, Banda campus, was performed using a TA.XT Express Connect Texture Analyser. Parameters such as skin strength, elasticity, hardness, springiness, and gumminess were evaluated.

**Figure 1:**
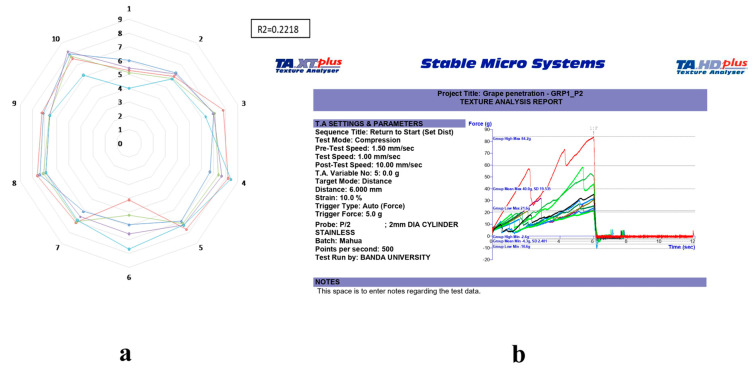
(**a**) Radar plot showing the sensory attributes (appearance, juiciness, texture, aroma, sweetness, hardness, springiness, gumminess, chewiness, and elasticity) of selected *M. longifolia* accessions. (**b**) Representative texture profile analysis obtained using a TA.XTplus Texture Analyser from Stable Micro Systems, illustrating the force–time curves used to determine mechanical properties of the samples.

Among the accessions, BM-6 exhibited the highest skin strength (139.14 ± 37.65 g) and hardness (136.59 ± 0.22), indicating a comparatively firmer texture. BM-7 showed the maximum elasticity (5.95 ± 0.98 mm), while BM-4 demonstrated the highest springiness (103.91 ± 0.66). BM-10 recorded the highest gumminess (50.11 ± 0.98) (Supplementary Table S1; Figure 1a).

Sensory evaluation using a 10-point hedonic scale identified BM-5 as the most preferred accession, exhibiting the highest scores for sweetness (8.18 ± 0.44) and aroma (8.10 ± 0.39). BM-1, although moderate in sweetness, showed superior texture firmness and stability, suggesting better shelf-life potential (Figure 1b).

Based on the overall texture and sensory performance, five accessions (BM-1, BM-4, BM-5, BM-6, and BM-10) were selected for further biochemical analysis.

### 2.2. The Biochemical Analysis of Primary Metabolites During Developmental Stages of Flowering in Selected Accessions of M. longifolia

Biochemical variations were investigated across five selected *M longifolia* accessions (BM-1, BM-4, BM-5, BM-6, and BM-10) during the transition from the immature flowering stage (IMF) to the mature flowering stage (MF). The results demonstrated significant accession-dependent differences in metabolite profiles.

**Figure 2:**
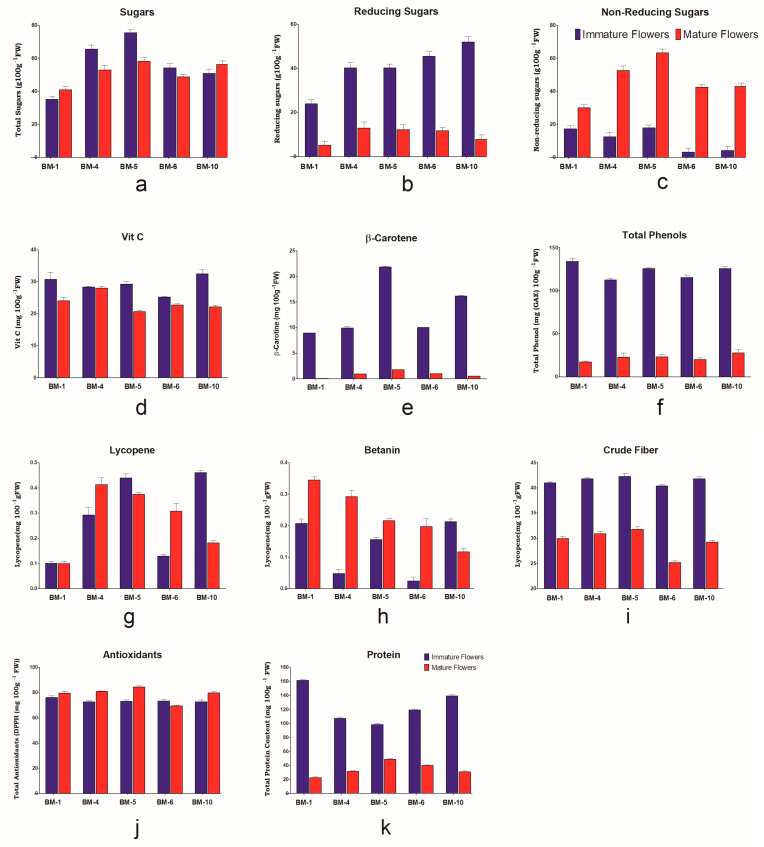
The biochemical analyses of primary metabolites in different accessions of *M. longifolia* flowers during developmental stages (from immature flowers to mature flowers) reveal significant variations in metabolite levels, reflecting dynamic biochemical changes during flower maturation. (**a**) Total Sugar, (**b**) reducing, (**c**) non-reducing sugar, (**d**) vitamin C, (**e**) beta-carotene, (**f**) phenol, (**g**) lycopene, (**h**) betanin, (**i**) crude fiber, (**j**) antioxidant properties, (**k**) total protein content.

With progression towards maturity, total sugar content exhibited an increasing trend, with BM-5 recording the highest accumulation, whereas BM-1 showed a marked decline. Reducing sugars predominated at the IMF, while non-reducing sugars were more abundant at the MF. Among the accessions, BM-10 showed the highest reducing sugar content at the IMF, whereas BM-6 recorded the highest levels at the MF (Figure 2a–c).

A declining trend was observed in vitamin C, β-carotene, total phenolic content (TPC), crude fiber, and total protein with advancing maturity. Conversely, lycopene content and antioxidant activity (DPPH assay) increased, indicating enhanced oxidative defense mechanisms in mature flowers (Figure 2d–g,i–k). Betanin content exhibited accession-specific variation, with an increasing trend in BM-5 and a slight reduction in BM-10 (Figure 2h).

Overall, BM-5 demonstrated superior performance, characterized by higher sugar retention, greater biochemical stability, and enhanced antioxidant and sensory attributes, suggesting its potential as a high-quality accession. In contrast, BM-1 exhibited comparatively lower biochemical stability and inferior quality-associated traits.

Based on these biochemical distinctions, BM-1 and BM-5 were selected for further in-depth analyses, including elemental profiling and gas chromatography, to elucidate compositional differences underlying quality traits.

### 2.3. Elemental Composition of M. longifolia Flower in Contrasting Accessions by ICP–MS

**Figure 3:**
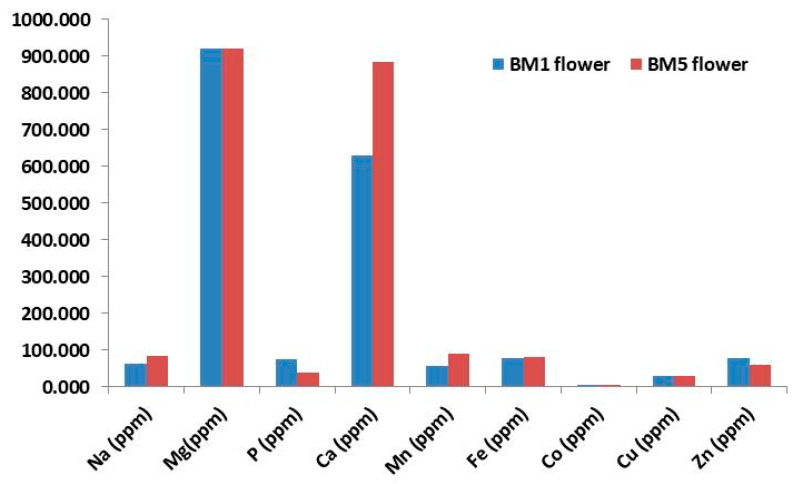
ICP–MS-based ionomic profiling of *M. longifolia* mature flowers in contrasting accessions BM-1 and BM-5.

In elemental profiling of two contrasting *M. longifolia* accessions (BM1 and BM5), MFs revealed distinct differences in mineral composition using only inductively coupled plasma mass spectrometry (ICP–MS). Among the elements detected, magnesium was present in higher and similar concentrations in both accessions. Meanwhile, calcium and iron concentration was higher in BM-5. Other trace elements, such as sodium, phosphorus, manganese, iron, cobalt, copper, and zinc, were found in comparatively lower amounts, but their relative distribution varied between accessions (Figure 3). These results demonstrate mineral diversity within *M. longifolia* accessions, suggesting that different accessions could serve as potential sources of specific micronutrients. Such variability detected by ICP–MS analysis provides valuable information for the nutritional value of different accessions.

### 2.4. GC–MS-Based Metabolite Profiling and Pathway Enrichment Analysis During Developmental Stages of Flowering in Contrasting M. longifolia Accessions

**Figure 4:**
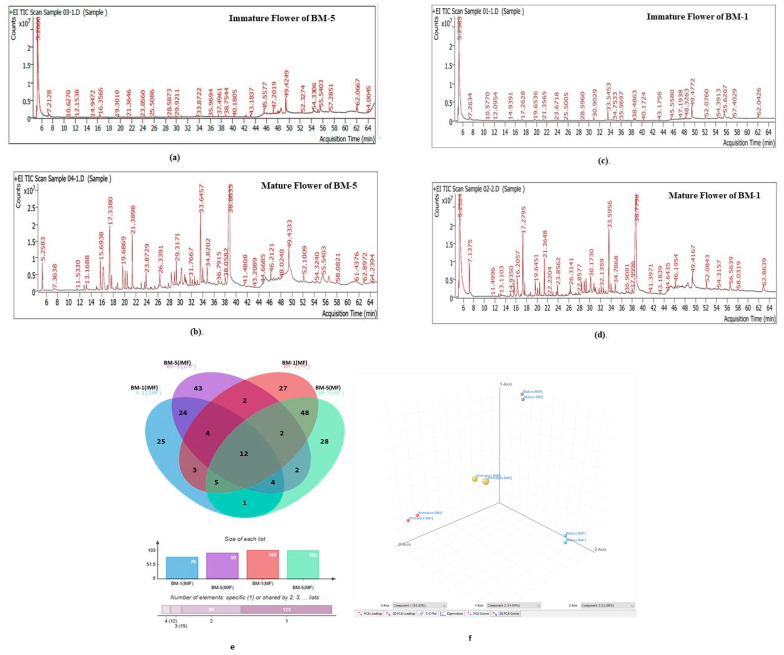
Gas chromatographic (GC) profile revealing accession—specific metabolites—in *M. longifolia* flowers (**a**) at immature flower stages of BM-5 and (**b**) mature flower stages of BM-5. (**c**) Immature Flower stages of BM-1. (**d**) Mature Flower stages of BM-1. (**e**) Comparison of metabolite distribution between developmental stages of *M. longifolia* flowers, highlighting stage-specific and common compounds by Venn diagram. (**f**) Principal component analysis (PCA) score plot showing metabolite clustering in *M. longifolia* flowers during developmental stages (IMF and MF) and accessions (BM-1 and BM-5).

GC–MS chromatograms of volatile and semi-volatile compounds in two contrasting *M. longifolia* accessions (BM-1 and BM-5) across IMFs and MFs identified 60, 94, 65, and 84 metabolites in BM-1IMF, BM-1MF, BM-5 IMF, and BM-5MF, respectively (Figure 4a–d and Supplementary Tables S2a,b and S3a,b). Venn analysis showed that BM-5MF had the highest number of unique metabolites (48), followed by BM-5IMF (43); meanwhile, BM-1IMF and BM-1MF contained only 25 and 27 unique compounds, reflecting a more limited biochemical spectrum. Twelve metabolites were common to all four groups, with additional partial overlaps indicating stage- and accession-specific divergence (Figure 4e). PCA explained 89.6% of the total variance (PC1: 63.09%, PC2: 14.54%, PC3: 12.08%), and the 3D plot revealed clear clustering by accession and developmental stage. BM-5MF separated strongly along PC1, while BM-1IMF grouped distinctly along PC2, confirming contrasting metabolic trajectories between the two accessions during flower development (Figure 4f).

**Figure 5:**
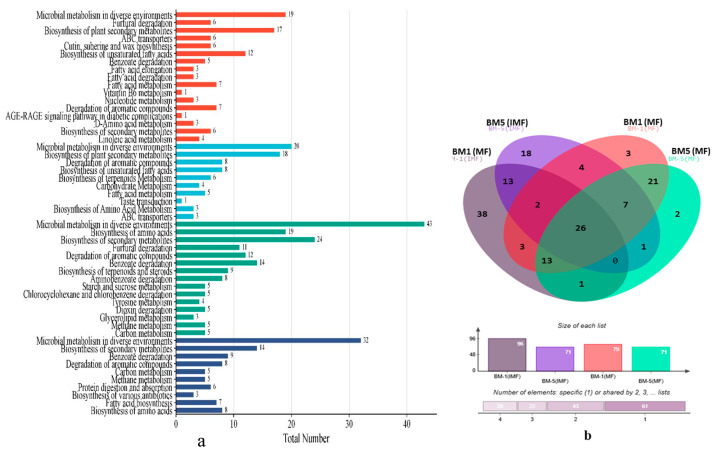
(**a**) KEGG pathway enrichment analysis of GC–MS–identified metabolites from IMFs and MFs of different *M. longifolia* accessions (BM-1 and BM-5). (**b**) Venn diagram-based comparison of stage–specific pathway analysis between developmental stages of different *M. longifolia* accessions.

Integrated KEGG pathway enrichment revealed clear metabolic variation among *M. longifolia* accessions and developmental stages. All four groups—BM-1IMF, BM-5IMF, BM-1MF, and BM-5MF—shared enrichment in pathways. BM-1MF showed the highest pathway diversity in ABC transporters, amino acid biosynthesis, and multiple degradation pathways, indicating a highly active metabolic state in the MF. BM-5 MF also shows pathway enrichment, particularly in microbial metabolism and secondary metabolite biosynthesis, although the total number of enriched pathways is slightly lower than in BM-1 MF. In contrast, the IMF groups (BM-1 IMF and BM-5 IMF) display relatively fewer enriched pathways, though they still include key processes such as furfural degradation and biosynthesis of plant secondary metabolites (Figure 5a).

Venn analysis showed 26 pathways common to all groups, representing the core metabolism. BM-1IMF was metabolically more distinct with 38 unique pathways, followed by BM-5 IMF (18 unique). In contrast, the MF displayed greater similarity, with only three and two unique pathways in BM-1MF and BM-5 MF, respectively, indicating metabolic convergence with maturation (Figure 5a,b). Overall, developmental stage exerted a stronger influence than accession, with BM-1 IMF being the most distinct and BM-1 MF showing the highest enrichment in pathways related to flavor, aroma, and physiological adaptation. These results underscore *M. longifolia* flowers as valuable sources of bioactive and aromatic compounds.

### 2.5. Hierarchical Clustering, Variable Importance in Projection (VIP) Score Analysis and Volcano Plot-Based Differential Metabolite Profiling of Flowering Developmental Stages in Contrasting Accessions of M. longifolia

**Figure 6:**
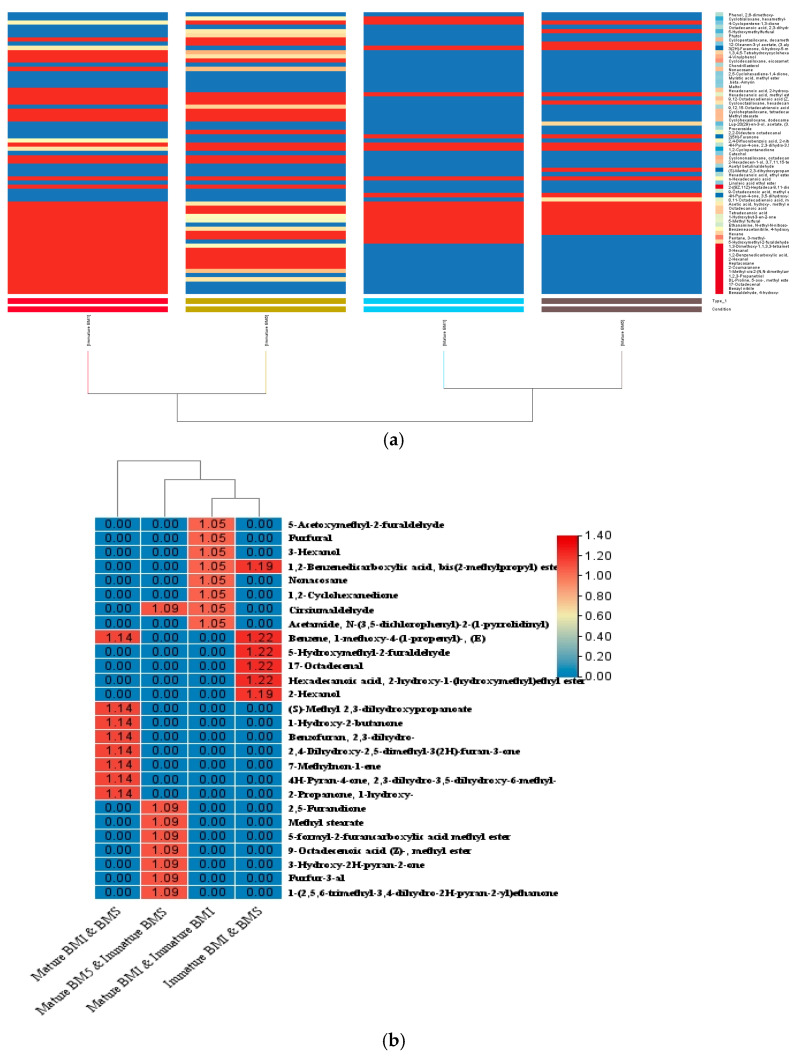
(**a**) Fingerprinting analysis of volatile metabolites in contrasting accessions of *M. longifolia* during flower developmental stages (IMF to MF), generated through GC–MS profiling. Distinct colors represent stage-specific accumulation and diversity of compounds. (**b**) VIP score plot derived from PLS-DA analysis highlighting key metabolites of taste and aroma.

The clustered heat-map dendrogram offers a global view of the volatile and semi-volatile metabolite profiles during the development of *M. longifolia* flower groups: BM1-IMF, BM5-IMF, BM1-MF, and BM5-MF. The dendrogram reveals that the primary separation among samples is based on developmental stage rather than accession. Mature flower samples (BM1-MF and BM5-MF) form one cluster, while immature samples (BM1-IMF and BM5-IMF) form another, indicating greater similarity in metabolite composition within each developmental phase (Figure 6a).

The VIP score-based heat-map revealed clear differences in aroma- and taste-related metabolites among the accessions. Although BM1 exhibited several high-VIP compounds, these were mainly associated with green and immature, including short-chain alcohols and aldehydes which contribute to aroma intensity but not necessarily to desirable taste. In contrast, BM-5 MF showed a greater contribution of furan, furanone, and pyran derivatives, which are known to impart sweet, caramel-like, and pleasant roasted aromas, indicating superior flavor quality. The BM5 samples, particularly at the immature stage, displayed fewer aroma-active compounds with lower VIP contributions, resulting in a comparatively mild sensory profile. Overall, the results demonstrate that BM5 MF possesses the most favorable aroma and taste characteristics, while BM1, despite higher discriminating metabolite abundance, exhibits inferior sensory quality compounds (Figure 6b and Supplementary Table S4). Overall, the combined clustering and VIP analyses demonstrate that developmental stage is the primary driver of metabolic differentiation in *M. longifolia* flowers.

The metabolomic profiling of *M. longifolia* flowers was performed using volcano plot analysis to identify statistically significant differences in metabolite accumulation. The analysis encompassed flower developmental stages (IMF and MF) and contrasting accessions (BM-1 and BM-5), thereby enabling the systematic evaluation of stage-dependent and accession-specific metabolic variations. In IMF, metabolites such as Hexadecanoic acid, 2-Hydroxy-1-(hydroxymethyl)ethyl ester, 17-Octadecenal, 1,2,3-Propanetriol, Benzyl nitrile, and 4-Hydroxybenzaldehyde showed significant differential accumulation.

In MF, higher levels of 5-Hydroxymethyl-2-furaldehyde (5-HMF), 5-Hydroxy-6-methyl-2,3-dihydropyran-4-one, 3H-Pyrazol-3-one, 2,4-Dihydro-4,5-dimethyl-, Benzofuran, 2,3-dihydro-, and 10E,12Z-Octadecadienoic acid were observed, whereas 4-Cyclopentene-1,3-dione, Phenol, 4-propyl-, 2-Furancarboxylic acid, and p-Hydroxystyrene were comparatively reduced.

**Figure 7:**
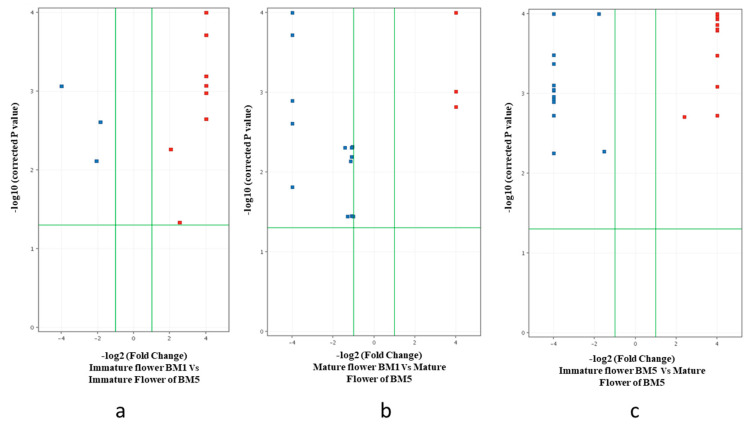
Volcano plots showing differential metabolite accumulation between immature and mature flowers of *M. longifolia* accessions BM-1 and BM-5. (**a**) Immature flowers of BM-1 vs. immature flowers of BM-5; (**b**) mature flowers of BM-1 vs. mature flowers of BM-5; (**c**) immature vs. mature flowers of BM-5. The *x*-axis represents log_2_(fold change) and the *y*-axis shows –log_10_(corrected *p*-value). Each point represents a metabolite detected by GC–MS. Red dots indicate significantly upregulated metabolites in BM-5, blue dots represent metabolites enriched in BM-1. Green vertical and horizontal lines indicate the thresholds for fold change (±2) and statistical significance (*p* < 0.05).

The transition from IMFs to MFs in BM-5 was also associated with increased accumulation of Hexadecanoic acid methyl ester, Methyl stearate, and 9,12-Octadecadienoic acid (Z,Z)- methyl ester, indicating developmental regulation of lipid-derived aroma and flavor metabolites (Figure 7a–c; Supplementary Table S5).

### 2.6. Regulation of Key Metabolites During Developmental Stages of Flowering in Contrasting Accessions of M. longifolia

GC–MS peak areas were used for relative metabolite quantification, and normalized values were applied to assess metabolic variation across floral developmental stages and contrasting *M. longifolia* accessions. GC–MS profiling revealed dynamic changes in sugars, fatty acid derivatives, furan compounds, and pyranone derivatives, indicating their roles in floral development, aroma formation, and accession-specific metabolic differentiation. These stage-dependent variations suggest coordinated biochemical regulation during flower maturation.

#### 2.6.1. Metabolic Regulation of Sugars and Their Derivatives During Developmental Stages of Flowering in Contrasting Accessions of *M. longifolia*

**Figure 8:**
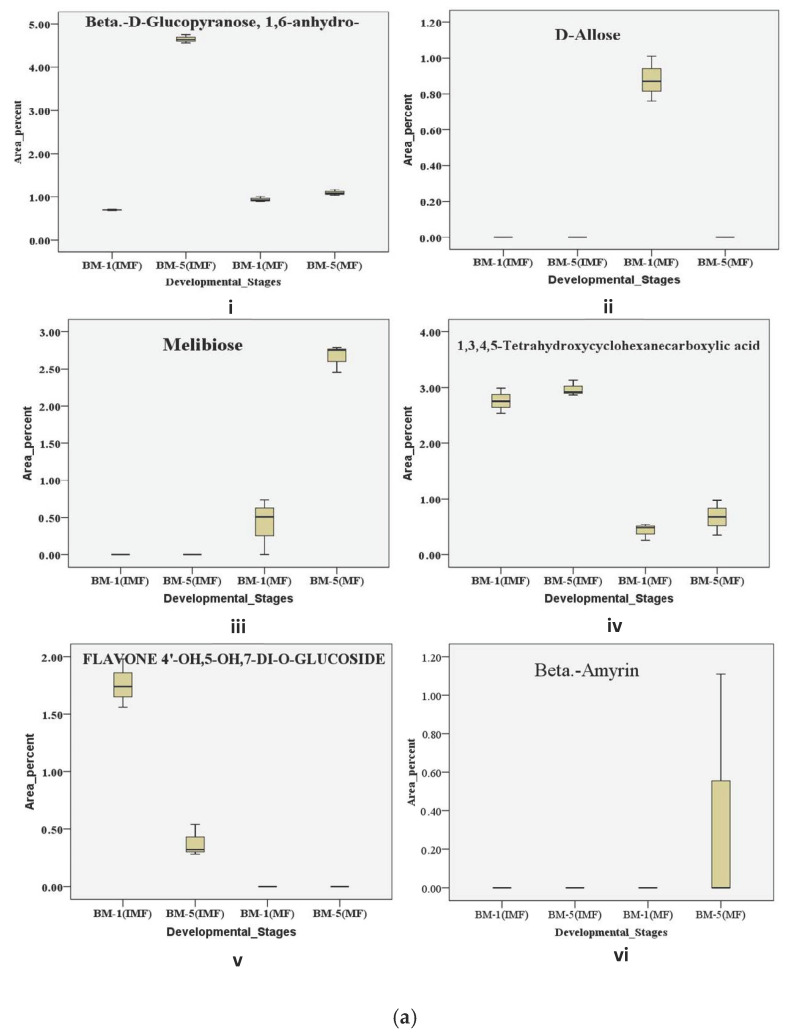

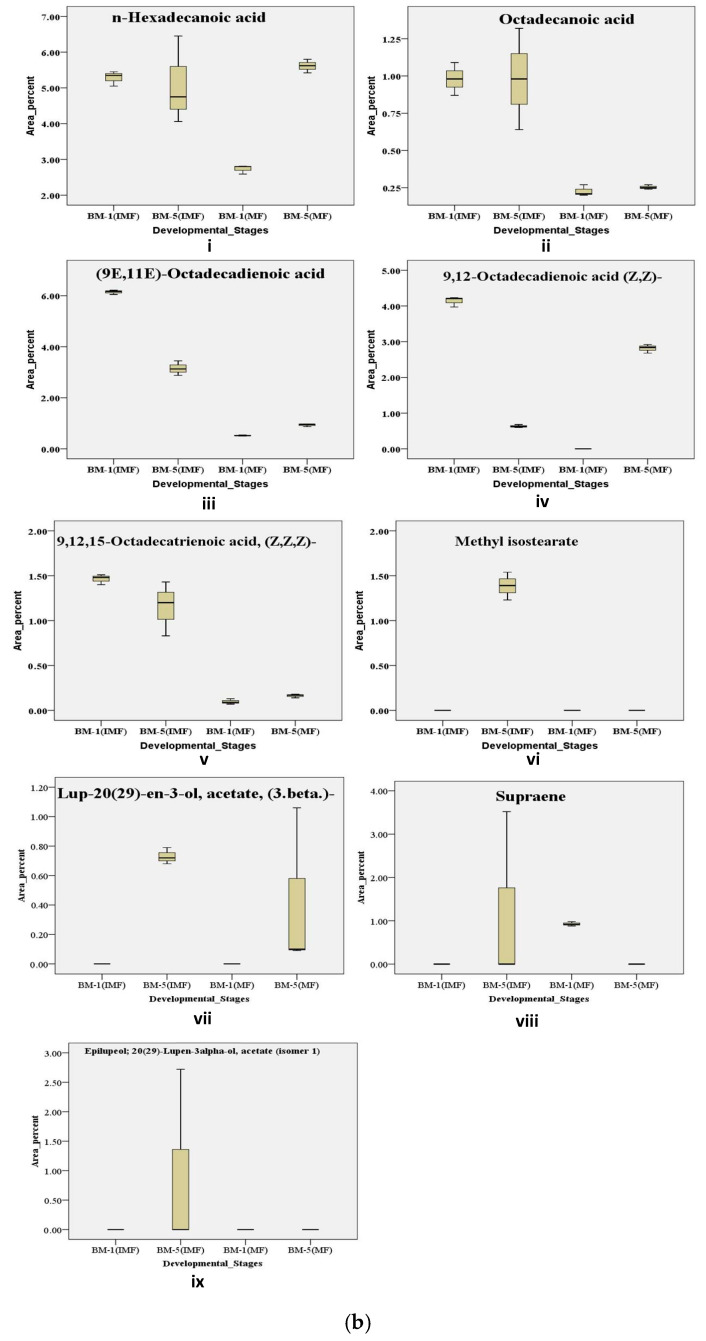

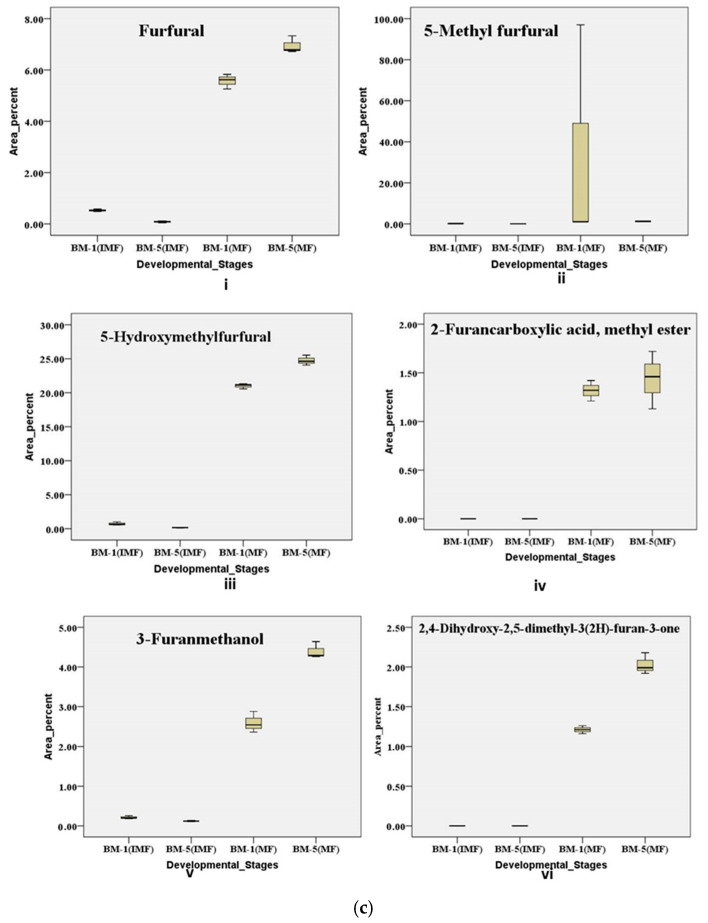

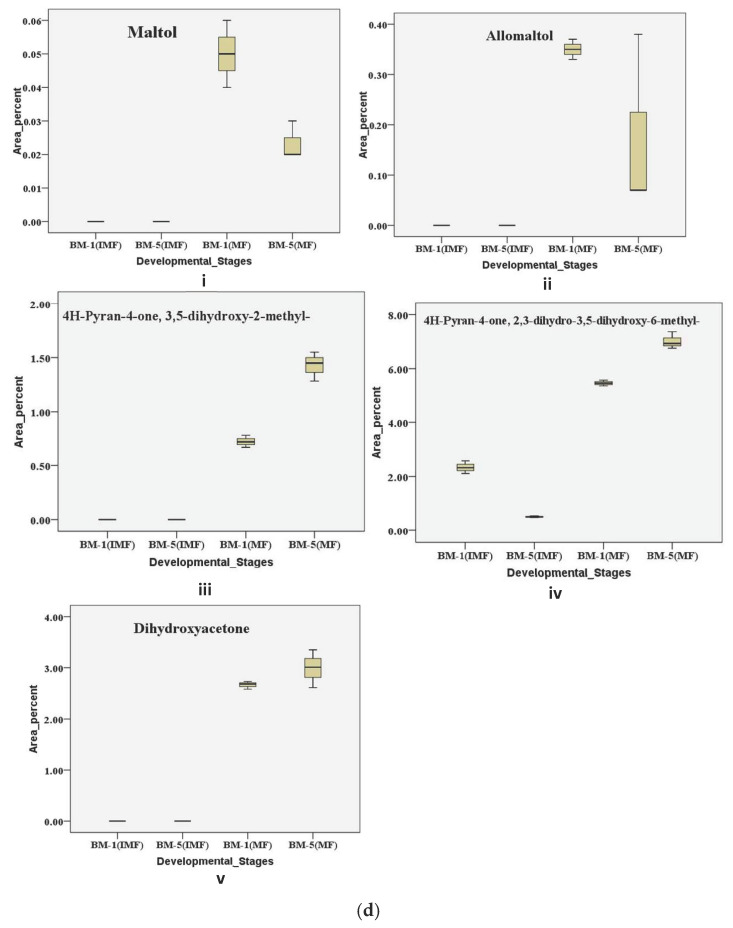
(**a**) Boxplot representation of key sugars and their derivative metabolites identified in *M. longifolia* flowers during developmental stages (IMF to MF) in contrasting accessions (BM-1 and BM-5). The plots show relative abundance of (**i**) Beta-D-Glucopyranose, 1,6-anhydro-, (**ii**) D-Allose, (**iii**) Melibiose, (**iv**) 1,3,4,5-Tetrahydroxycyclohexane carboxylic acid, (**v**) Flavone-4′-O-β-D-glucoside, and (**vi**) Beta-Amyrin. Data illustrate stage-specific and accession-specific metabolite accumulation patterns. (**b**) Boxplot representation of key fatty acid-derived and terpenoid metabolites identified in *M. longifolia* flowers during developmental stages in two contrasting accessions (BM-1 and BM-5). The plots show the presence of (**i**) Hexadecanoic acid, (**ii**) Octadecanoic acid, (**iii**) (9E,11E)-Octadecadienoic acid, (**iv**) 9,12-Octadecadienoic acid (**v**) 9,12,15-Octadecatrienoic acid (**vi**) Methyl isostearate, (**vii**) Lup-20(29)-en-3β-ol acetate, (**viii**) Suprane, and (**ix**) Epilupeol [20(29)-lupen-3α-ol] acetate. The boxplots illustrate stage-specific and accession-specific patterns of metabolite accumulation during flower development. (**c**) Boxplot representation of key furan-derived metabolites identified in *M. longifolia* flowers during developmental stages in two contrasting accessions (BM-1 and BM-5). The plots depict the presence of (**i**) furfural, (**ii**) 5-methylfurfural, (**iii**) 5-hydroxymethylfurfural, (**iv**) 2-furan carboxylic acid, (**v**) 3-furanmethanol, and (**vi**) 2,4-dihydroxy-2,5-dimethyl-3(2H)-furan-3-one. The boxplots illustrate stage-specific and accession-specific variations in metabolite accumulation during flower development. (**d**) Boxplot representation of Pyranone and its derived metabolites identified in *M. longifolia* flowers during developmental stages in two contrasting accessions (BM-1 and BM-5). The panels represent the relative abundance of (**i**) Maltol, (**ii**) Allomaltol, (**iii**) 4H-Pyran-4-one, 3,5-dihydroxy-2-methyl-, (**iv**) 4H-Pyran-4-one, 2,3-dihydro-3,5-dihydroxy-6-methyl-, and (**v**) dihydroxy acetone. The boxplots illustrate stage-specific and accession-specific variations in metabolite accumulation during flower development.

Sugar metabolism showed clear developmental and accession differences. β-D-Glucopyranose, 1,6-anhydro-, also known as levoglucosan, was higher in BM-5 IMF (4.65) than BM-1 (0.70) but declined at maturity. D-Allose was detected only in BM-1 MF (0.88), while Melibiose accumulated in BM-5 MF (2.66), indicating accession-specific roles in maturation. 1,3,4,5-Tetrahydroxycyclohexane carboxylic acid (Quinic acid) was present across all stages, with higher levels (~3.0%) in IMFs of both accessions and lower (~1.0–1.5%) at maturity. Flavone 4′-OH,5-OH,7-di-O-glucoside occurred only in the IMF, being higher in BM-1 (1.76). Beta-Amyrin was absent in the IMF and BM-1 MF but appeared specifically in BM-5 MF (~0.5–1.0%) (Figure 8a).

#### 2.6.2. Contributionof the Metabolic Regulation of Fatty Acid Derivatives During Developmental Stages of Flowering in Contrasting Accessions of *M. longifolia*

Lipid profiling showed stage- and accession-specific shifts. n-Hexadecanoic acid (palmitic acid) was consistently abundant, especially in BM-1(IMF: 5.28) and BM-5(MF: 5.61), implying structural roles. Octadecadienoic acid was higher in the MF of BM1 while absent at BM-5 mature stage. (9E,11E)-Octadecadienoic acid peaked in BM-1(IMF: 6.15), while 9,12-Octadecadienoic acid (linoleic acid) was found in BM-1(IMF: 4.14) and BM-5(MF: 2.81). 9, 12, 15 Octodecatrienoic acid was present at the IMF, and Methyl isostearate was unique to BM-5(IMF: 1.39), suggesting active fatty acid methylation. Among terpenoid-derived compounds, Lup-20(29)-en-3-ol, acetate (3β-), also known as Lupeol acetate, Supraene, and Epilupeol, were absent at the BM-1 IMF but presentin BM-5 IMF, with Lup-20(29)-en-3-ol, acetate (~2.0%) and Epilupeol (~1.5%) showing abundance, while Supraene reached ~1.5%. At maturity, their levels declined sharply, with only low amounts of Lup-20(29)-en-3-ol, acetate (~1.0%) and Supraene (~1.0% in BM-1 mature) detected, whereas Epilupeol was undetectable, indicating their preferential roles during IMF development (Figure 8b).

#### 2.6.3. Metabolic Regulation of Furan and Furan Derivatives During Developmental Stages of Flowering in Contrasting Accessions of *M. longifolia*

Furan-related volatiles increased during maturation. Furfural and 5-Hydroxymethylfurfural (HMF) were highly elevated in BM-5(MF: 6.94 and 24.74), respectively, indicating sugar dehydration and oxidative metabolism. 5-Methyl furfural was highest in BM-1(MF: 33.02), while 2 Furancorboxylic acid, Methyl ester,3-Furanmethanol and 2,4 Dihydroxy-2-5 dimethyl-3(2H)-Furan-3-one (also known as Furaneol) derivatives appeared predominantly in mature BM-5 flowers, suggesting a link to floral scents and late-stage stress responses (Figure 8c).

#### 2.6.4. Contribution of Metabolic Regulation of Pyranone and Its Derivative During Developmental Stages of Flowering in Contrasting Accessions of *M. longifolia*

During the developmental progress of *M. longifolia* flowers, pyranone derivatives showed stage-specific patterns. Maltol and Allomaltol were absent at the IMF (BM-1) but accumulated significantly at MF, with higher abundance in BM-1 MF compared to BM-5.4H-Pyran-4-one, 3,5-dihydroxy-2-methyl, a hydroxylated derivative of Maltol, was undetectable in IMF but increased steadily, reaching maximum levels at BM-5 MF. In contrast, 4H-Pyran-4-one, 2,3-dihydro-3,5-dihydroxy-6-methyl (hydroxydihydromaltol) was higher in BM-1 IMF, decreased at BM-5, and rose again at MF. Dihydroxyacetone (DHA), absent in IMF, appeared at the MF of BM-1 and remained significant in BM-5, reflecting active carbohydrate metabolism (Figure 8d). 

## 3. Discussion

### 3.1. Texture and Sensory Analysis of Flowers Collected from Different Accessions of M. longifolia

The pronounced sensory and textural diversity observed across *M. longifolia* accessions highlights the underlying texture and sensory complexity of the different accessions. Variations in firmness, elasticity, and chewiness resemble patterns previously documented in grape, berries, dates, and apple [9,10,11,12,13], indicating that mechanical properties of floral tissues are strongly genotype-dependent and likely governed by differential cell-wall organization and moisture retention capacity. Such diversity underscores the importance of identifying accessions aligned with consumer-preferred softness and sweetness for optimized processing applications.

### 3.2. The Biochemical Analysis of Primary Metabolites in Developmental Stages of Flowering in Accessions of M. longifolia

The biochemical analysis of *M. longifolia* flowers across accessions (BM-1, BM-4, BM-5, BM-6, BM-10) showed significant developmental variation. BM-5 had the highest total sugar content in mature flowers, indicating greater sweetness, consistent with earlier findings [14,15]. Reducing sugars were higher in the IMF, indicating intense metabolic activity during early development, whereas non-reducing sugars increased at the MF, reflecting the conversion and accumulation of sucrose for reproductive and storage functions. Developmental regulation of sugar metabolism and inter-conversion between sugar forms has been widely reported in plants [16], and Mahua flowers exhibit dynamic carbohydrate changes during maturation [17], with sugars also contributing to floral metabolite biosynthesis [16,17,18]. BM-5 total sugar content was nearly similar to previously reported Mahua [17] but higher than juicy fruit like ripened grapes [19,20], supporting its superior palatability. Total protein content peaked in the IMFofBM-1 and BM-5 MF, aligning with ~6.37% protein reported for *M. longifolia* [21,22]. β-carotene was highest in IMF BM-5 and decreased with the MF, similar to observations in vegetable [23]. Lycopene levels were high in IMFBM-5 andBM-4MF, with levels comparable to values in beetroot and tomato [24,25], highlighting the functional food potential of *M. longifolia*.

Betanin was present in both IMFs and MFs of BM-4 and BM-5 and was similar to levels in pitaya and garambullo [26,27], supporting its use as a natural colorant. A stage-dependent shift in pigments, also seen in *Tageteserecta* [28], suggests a common developmental trend across species. Vitamin C content varied by tissue and stage, with maximum levels in BM-4, and BM-5 MF and IMF BM-10 matching earlier reports [16,29]. DPPH-based antioxidant activity was highest among the IMF, with BM-1 showing comparatively higher antioxidant capacity, whereas BM-5 exhibited the highest antioxidant activity among the MF. The antioxidant levels in IMF BM-1 and MF BM-5 were comparable to those reported for red grapes and grape pulp [30,31], indicating strong health-promoting potential. Crude fiber and Phenol were highest in all IMFs, aligning with previously reported values [14,32,33]. Although phenolics and β-carotene were higher in the IMF, the increased antioxidant activity observed in the MF may be attributed to the cumulative and synergistic effects of multiple bioactive compounds, including lycopene, betanin, total sugars, and other secondary metabolites that increase during maturation. Additionally, changes in the chemical structure, extractability, and bioavailability of antioxidant compounds during flower development may enhance radical scavenging capacity in mature flowers. The contribution of non-phenolic antioxidants and Maillard reaction products formed during maturation may also play a role in increasing antioxidant activity [34,35]. Furthermore, the antioxidant assays such as DPPH measure the overall radical scavenging capacity, which reflects the combined effect of all antioxidants rather than individual compounds.

Thus, both accession and developmental stage significantly shape the nutritional and phytochemical profile of *M. longifolia*. Accessions such as BM-5 and BM-4, with superior sweetness, pigments, antioxidants, and nutrients, show strong potential for value-added products, natural supplements, and functional food applications.

### 3.3. Elemental Composition of M. longifolia Flower in Contrasting Accessions by ICP–MS

Mineral diversity revealed by ICP–MS emphasizes the nutritional importance of *M. longifolia*, as many accessions contained higher elemental levels than Brassica or Nasturtium flowers [36,37]. Sensory modulation by minerals is well-established: Na enhances sweetness and suppresses bitterness via ENaC-mediated pathways [38,39], while Ca and Mg influence taste through TRPM5 and TRPV1 activation [40,41] and contribute to kokumi perception [41]. The presence of Fe- and Zn-associated metallic notes [42,43] may further shape accession-specific palatability. These mineral–taste interactions demonstrate that the flavor quality of *M. longifolia* flowers is not solely sugar-driven but is also influenced by ionic balance [43,44]. Their high essential mineral content additionally reinforces the species’ value as a functional food resource.

### 3.4. GC–MS-Based Secondary Metabolite Profiling and KEGG Pathway Mapping in Developing Flowers of Contrasting M. longifolia Accessions (BM1 and BM5)

GC–MS profiling of *M. longifolia* accessions BM-1 and BM-5 across immature and mature flower stages revealed developmental and accession-specific differences, with BM-5—particularly at maturity—showing the highest VOC diversity and the greatest number of unique metabolites. BM-1 displayed a more conserved profile, while both accessions shared a core set of compounds. IMFs were richer in alcohols and ethers, whereas MFs contained more small sugars, ketones and esters, with terpenes present throughout, consistent with the sesquiterpene and (E,E)-farnesol dominance previously reported for *M. longifolia* [18]. Similar ranges of VOC richness have been observed in other flowers, including roses [45], *Luculia pinceana* [46], *Tillandsia* species [47,48], and *Lycoris taxa* [49], as well as in other metabolite-rich plants such as *Asparagus* bean seeds [50].

Integrated VOC and KEGG pathway analyses further revealed clear metabolic reprogramming during floral development. The IMF showed enrichment in alkaloid, terpenoid, phenyl propanoid, and carbon metabolism pathways, while the MF shifted toward hormone, terpenoid, and steroid biosynthesis. These developmental trends reflect the functional roles of floral VOCs in defense and pollinator signaling [51,52,53], and align with established MEP/MVA pathway contributions to terpenoid and phenyl propanoid formation [54,55,56]. Sulfur- and nitrogen-containing volatiles also support ecological communication [49]. Overall, BM-5 exhibits greater metabolic plasticity and VOC diversification, highlighting its strong potential for aromatic, industrial, ecological, and therapeutic applications.

### 3.5. Integrated Hierarchical Clustering, VIP Scores, and Volcano Plot Analyses Reveals Developmental Regulation of Key Metabolites in Contrasting M. longifolia Accessions

The integrated metabolomic profiling of *M. longifolia* flowers clearly demonstrates that floral development drives a strong and structured biochemical shift, separating IMFs and MFs across clustering, VIP, and volcano analyses. This developmental transition aligns with pathways that enhance aroma formation, nutritional stability, and processing suitability—traits vital for fermentation industries, flavor extractions, nutraceutical applications, and broader bio-based utilizations. A key outcome of maturation is the marked accumulation of furan derivatives such as furfural, 5-HMF, and Methyl furfural, most prominently in BM-5. These Maillard-derived compounds confer roasted, caramel-like, and sweet aromatic, making mature flowers naturally richer in flavor precursors [57,58,59]. Their elevated presence in BM-5 indicates strong suitability for high-aroma distillates and enhanced fermentation complexity [60,61]. Beyond sensory functions, compounds such as 5-HMF exhibit antioxidant, anti-allergic, anti-inflammatory, anti-hypoxic, anti-sickling, and anti-hyperuricemic effects and functional bioactive properties, supporting their relevance in natural preservatives and plant-based flavor enhancers [62,63,64]. The strong anti-inflammatory effects of 5 HMF in LPS-activated macrophages is due to it suppressing MAPK, NF-κB, and Akt/mTOR signaling pathways. It shows promise as a bioactive functional food with therapeutic potential [65].

IMFs, in contrast, show higher levels of fattyacid derivatives and long-chain aldehydes—particularly in BM-5—indicating the early activation of lipid metabolism essential for membrane stability and developmental readiness. Fatty acid esters like methyl stearate and linoleic acid methyl ester have industrial significance as precursors for bio-lubricants, emulsifiers, and surfactants, widening *M. longifolia*’s potential into pharmaceutical applications [66,67]. Such stage-specificity highlights the importance of selective harvesting for maximizing targeted metabolite yields.

Terpenoid-associated metabolites (Lupeol acetate, Epilupeol, Supraene, β-Amyrin) dominate the IMF of BM-5, reinforcing their pharma-industrial value [68]. These compounds—known for anti-inflammatory, antimicrobial, wound-healing, and antioxidant properties—strongly support the use of the IMF for nutraceutical and herbal formulations [69,70,71,72]. Their decline at maturity further underscores the critical timing required to capture peak bioactivity. Nitrogenous and phenolic volatiles, including benzyl nitrile and 4-hydroxybenzaldehyde, were also significantly elevated in BM-5, suggesting greater metabolic investment in aroma and stress-responsive compounds [73].

Distinct stage- and accession-linked differences were also evident in sugars, fatty acids, and related derivatives. Sugar-derived metabolites also show meaningful accession-specific patterns. Sugars such as β-D-Glucopyranose, D-Allose, and Melibiose displayed characteristic patterns linked to energy regulation and osmotic balance [74,75]. Levo glucosan and Melibiose indicate differential carbohydrate mobilization and stress response, while the detection of D-Allose in BM-1 MF is noteworthy due to its reported insulin-sensitizing, anti-inflammatory, and cyto-protective activities [75,76,77,78]. Quinic acid, consistently detected across stages, contributes as a precursor to chlorogenic acids and enhances the nutritional value of *M. longifolia*-based foods and beverages [79].

Flavonoid glycosides like flavone 4′-OH,5-OH,7-di-O-glucoside were enriched in BM-1 IMF, indicating roles in defense, pigmentation, and antioxidant activity [80]. Fatty acids, including Palmitic acid and Poly-unsaturated species, showed dynamic modulation supporting membrane stability and signaling [81,82]; meanwhile, methyl isostearate in BM-5 IMF suggests active ester biosynthesis pathways [83].

Furan and pyran derivatives—furfural, HMF, Furaneol, Maltol, Allomaltol, and 4H-Pyran-4-one compounds—were especially abundant in BM5MF, reflecting intensified sugar degradation and Maillard-reaction activity [84,85,86,87]. Mechanistically, MF conditions favor furan and furanone formation through sugar–amino acid interactions, whereas IMFs suppress these reactions due to lower moisture or altered pH. BM-5 MF showed the strongest Maillard signature, suggesting superior potential for aroma enhancement [88], while BM-1 MF maintained higher native sugars and flavonoids, balancing primary and secondary metabolism.

Overall, IMFs favored bioactive secondary metabolites (flavonoids, cyclitols, polyunsaturated fatty acids), whereas MFs enhanced sugars, triterpenoids, and Maillard products. BM-5 consistently displayed stronger accumulation of furans and triterpenoids, reflecting higher metabolic capacity. The interplay of developmental stage, accession genetics, and biochemical reprogramming clearly shapes *M. longifolia*’s aroma, nutritional, and industrial potential.

From a processing viewpoint, these findings provide a practical blueprint for value-addition. IMFs are optimal for triterpenoid-rich nutraceuticals, while MFs offer superior substrates for flavor extraction, fermentation-based beverages, and aroma standardization. Accession-specific metabolic fingerprints enable targeted extraction strategies for furans, esters, and terpenoids and facilitate the development of standardized, quality-controlled *M. longifolia*-based flavor and nutraceutical products. Together, these insights support the design of accession-specific valorization pipelines that align metabolite strengths with industrial applications and market-driven product innovation.

## 4. Materials and Methods

### 4.1. Collection of Immature and Mature Flowers of M. longifolia

**Figure 9:**
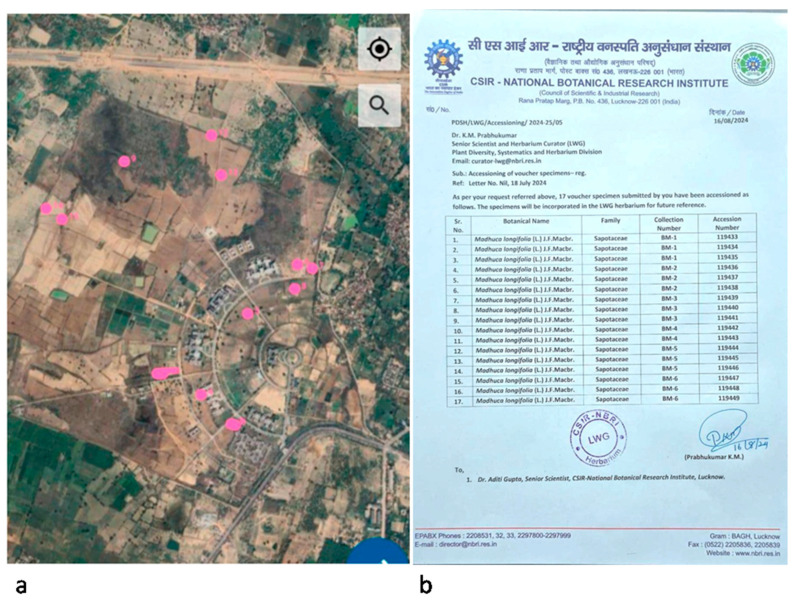

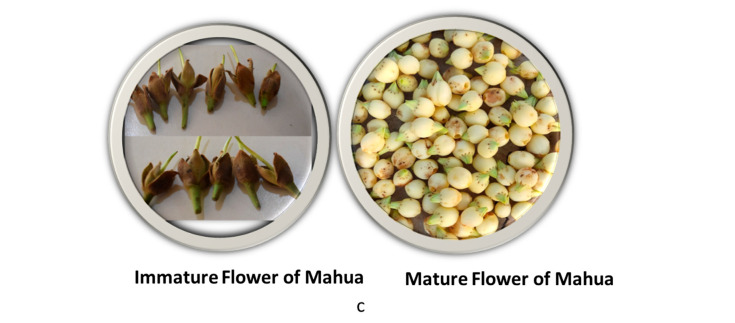
(**a**) Location of the *M. longifolia* accessions (BM-1 to BM-13) in the orchard at Banda University of Agriculture and Technology (BUAT), Banda, Uttar Pradesh, India. (**b**) The authenticity of *M. longifolia* accessions (BM-1 to BM-6) was confirmed by the National Botanical Research Institute (NBRI), Lucknow, and voucher specimens were deposited for future reference. (**c**) Immature and mature flowers of *M. longifolia*.

*M. longifolia* orchards located within the Banda University of Agriculture and Technology (BUAT), Banda campus, Uttar Pradesh, were selected for the present study (Figure 9a). BM-1 to BM-13 represent thirteen different accessions of *M. longifolia* collected from the BUAT orchard. Six *M. longifolia* accessions were taxonomically verified and authenticated by the National Botanical Research Institute (NBRI), Lucknow (Figure 9b). Immatureand mature flowers were directly collected during the morning hours from the campus orchards. Freshly collected samples were then processed for further experimental analysis (Figure 9c).

### 4.2. Sensory and Instrumental Texture Analysis

A sensory evaluation of different *M. longifolia* BM-1–BM-13 flowers was performed using a 9-point hedonic scale by a panel of seven judges. Samples, coded with three-digit identifiers, were assessed for appearance, juiciness, texture, aroma, and sweetness. Each judge evaluated ten anonymous samples, expressing preferences from “Dislike Extremely” (1) to “Like Extremely” (9) [89]. Texture measurements of *M. longifolia* flowers from 13 accessions were conducted using a TA.XT Express Connect Texture Analyzer (Stable Micro Systems Ltd., Godalming, Surrey, UK) with a 10 kg load cell and Express Connect Lite software (version 2.0). Tests in compression and tension were performed at a speed range of 0.1–10 mm/s, with a maximum testing pressure of 210 mm. Five berries from each accession were tested at room temperature (25 °C) using a P/2 probe (2 mm cylinder). Test parameters included pre-test speed (1.50 mm/s), test speed (1.00 mm/s), post-test speed (10.00 mm/s), distance (6.00 mm), strain (10%), and trigger force (5 g). Skin strength and elasticity were derived from the force–time curve.

### 4.3. Biochemical Analysis of Primary Metabolites

Fresh flowers of *M. longifolia* from selected accessions were analyzed at IMFs and MFs for primary metabolites.

#### 4.3.1. Carbohydrates

Total carbohydrates were estimated using phenol–sulfuric acid, with absorbance at 490 nm. Reducing sugars were determined by the method using alkaline copper tartrate and arsenomolybdate reagents, with absorbance at 510 nm [90,91,92]. Non-reducing sugars were calculated as the difference between total and reducing sugars.

#### 4.3.2. Protein

Protein was extracted in Tris-HCl buffer (pH 7.5) and estimated by the method using Folin’s phenol reagent, with BSA as the standard [93].

#### 4.3.3. Ascorbic Acid

This was quantified by titration with 2,6-dichlorophenol indophenol using oxalic acid extracts [94,95].

#### 4.3.4. Antioxidant Activity

Antioxidant activity was measure using the DPPH radical scavenging assay [96], with absorbance recorded at 517 nm after incubation in the dark.

#### 4.3.5. β-Carotene

This was extracted with acetone–petroleum ether and quantified spectro-photometrically at 452 nm [97].

#### 4.3.6. Total Phenolic Content

This was estimated using Folin–Ciocalteu reagent in alkaline medium with catechol as the standard [98].

### 4.4. Mineral Analysis by ICP-MS

Mineral content in *M. longifolia* flowers was quantified using inductively coupled plasma mass spectrometry (ICP-MS; Model: Agilent ICP-MS 7850)(Agilent, Santa Clara, CA, USA). Flower samples from each stage and accession were washed, oven-dried at 60 °C, and ground into a fine powder. A total of 0.5 g of the sample was digested with concentrated nitric acid (HNO_3_) and hydrogen peroxide (H_2_O_2_) using a microwave digestion system (Model: Microwave Digestion System, Multiway GO Plus (Anton Paar, Graz, Austria), followed by dilution with deionized water. Digested samples were analyzed using ICP-MS, with calibration performed using certified multi-element standards and internal standards to correct for instrumental drift. Each sample was analyzed in triplicate, and mineral concentrations were expressed as mg/kg dry weight. Blanks and standard reference materials were included to ensure accuracy and reproducibility. Statistical analysis was performed to compare mineral profiles in different accessions [99].

### 4.5. GC-MS Analysis

#### 4.5.1. Extraction of Volatile Compounds

Each sample were accurately weighed 5 g ± 0.0005, transferred to 50 mL centrifuge tube, 25 mL Methanol: n-Hexane (99: 1% *v*/*v*) was added, then they were vortexed at same moment for 60 min using a NeuationiSwixMV Multi Vortex Mixer at 2500 rpm, and then centrifuge at 10,000 rpm at 10 °C for 10 min. Then transfer 5 mL of supernatant into a 20 mL glass tube, evaporate the solution completely at 40 °C, reconstitute it up to 2 mL with methanol, and then subject it to filtering through a 0.45 µm PVDF filter and use it for analysis purposes.

#### 4.5.2. GC-MS Analysis and Data Interpretation

The GC-MS analysis was performed on an Agilent 8890 gas chromatograph coupled with an Agilent 5977B MS detector (GC-MS) (Agilent, Santa Clara, CA, USA). The separation of volatile compounds was carried out on a DB-5MS capillary column (30 m × 0.32 mm × 0.25 μm film thickness). Helium (purity > 99.999%) was employed as the carrier gas, with a constant flow rate of 1.2 mL/min. The injection volume was 1 µL. The temperature of the injection port was set to250 °C. The pulsed splitless mode was used. The oven temperature was set to 45 °C at the initial stage for 3 min, then increased to 200 °C at 5 °C/min, held for 10 min, followed by an increase to the final temperature of 250 °C at a rate of 40 °C/min and held for 20 min. The mass spectrometry was conducted with the ionization mode of EI with an electron energy of 70 eV. The temperature of the ion source and quadrupole were set at 280 °C and 150 °C, respectively. Full scan mode was applied, with a mass scan range of 35–800 atomic mass unit (amu). Data processing for compound identification was performed using the Wiley Registry 12th Edition/NIST 2020 Mass Spectral Library by MassHunter Workstation Qualitative Analysis Version 10.0.10305.0 (Agilent Technologies, Palo Alto, CA, USA).

Volatile compounds were identified by comparing retention indices (RIs), calculated using a C7–C30 n-alkanes standard mixture, and mass spectral fragmentation patterns with corresponding Kovats indices and mass spectra from the Wiley Registry 12th Edition/NIST 2020 library, using MassHunter Workstation Qualitative Analysis software (Version 10.0), as well as from published literature data [100,101,102].

### 4.6. Statistical, KEGG, Variable Importance in Projection (VIP), and Data Analysis

The study was conducted using a completely randomized design. Statistical analyses, including ANOVA, were performed using OPSTAT (CCSHAU, Hisar, India) and SPSS software-30. GC–MS data were processed to identify and quantify metabolites using Agilent MassHunter. The resulting peak area matrix was normalized and scaled, followed by partial least squares discriminant analysis (PLS-DA 2025) to assess group separation. Variable importance in projection (VIP) scores were then calculated from the PLS model based on variable weights and explained variance, with compounds showing VIP values greater than 1 considered significant contributors to sample differentiation [103]. Differentially expressed metabolites were mapped to the KEGG database for pathway enrichment analysis. KEGG enrichment was performed with a significance threshold of FDR q ≤ 0.05 [104,105].

## 5. Conclusions

This work provides the first integrated, multi-trait characterization of *M. longifolia* flower accessions by combining texture profiling, nutritional and phytochemical analysis, mineral composition, and comprehensive GC–MS-based metabolomics across developmental stages. Such a holistic dataset has not been reported previously for *M. longifolia*. IMFs were dominated by fattyacid esters, aldehydes, flavonoid glycosides, and terpenoids, while MFs transitioned toward carbohydrate-derived volatiles, particularly furans, pyranones, esters, and other aroma-active compounds. Among all accessions, BM-5 displayed the most diverse and responsive metabolic profile, characterized by higher sugars, carotenoids, and a broader array of volatiles, including key fermentation and aroma markers such as melibiose and furfural, 5-HMF, and furaneol, respectively. Its enrichment in several biologically relevant metabolites further strengthens its suitability for food, aromatic, and health-oriented applications. Multivariate and pathway analyses consistently supported BM-5 as the most metabolically flexible accession.

Overall, this integrated analysis establishes clear accession-specific strengths that can guide targeted utilization. BM-5 is best aligned with flavor, fermentation, distillation, and nutraceutical industries, while BM-1 and BM-4 provide valuable profiles for antioxidant- and protein-rich formulations. These insights address the current lack of accession-specific guidelines for *M. longifolia* valorization and create new opportunities for cultivar selection, processing optimization, and sustainable commercialization of this culturally and economically important tree species.

## Data Availability

All data generated or analyzed during this study are included in this published article (and its Supplementary Materials).

## Supplementary Materials

The following supporting information can be downloaded at: https://www.mdpi.com/article/10.3390/molecules31111977/s1, Supplementary Table S1: Texture analysis and Sensory evaluation of Mahua Flower collected from different accession from BUAT Campus; Supplementary Table S2: (a) List of secondary metabolites compound identified by GS-MS in Immature Flower of BM-1; (b) List of secondary metabolites compound identified by GS-MS in mature Flower of BM-1; Supplementary Table S3: (a) List of secondary metabolites compound identified by GS-MS in Immature Flower of BM-5; (b) List of secondary metabolites compound identified by GS-MS in mature Flower of BM-5; Supplementary Table S4: VIP Score; Supplementary Table S5: Differential metabolites identified by volcano plot analysis comparing immature and mature flower stages between contrasting *M. longifolia* accessions (BM1 and BM5).

## Abbreviations

The following abbreviations are used in this manuscript:

KEGG	Kyoto Encyclopedia of Genes and Genomes
GC–MS	Gas Chromatography–Mass Spectrometry
PCA	Principal Component Analysis
VIP	Variable Importance in Projection
