# Supplementary material for: Chemo-Diversity Landscape Using Physico-Biochemical, Elemental, and Metabolic Profiling in Different Stages and Accessions of Madhuca longifolia Flowers for Unveiling Their Processing Value and Utilization
Source: Molecules. 2026 Jun 5;31(11):1977. doi: 10.3390/molecules31111977 (PMC13258767; doi:10.3390/molecules31111977)
Supplement: Supplementary file 1 [file molecules-31-01977-s001.zip › Supplementary table S1-texture analysis.pdf]

Supplementary Table S1: Texture analysis and Sensory evaluation of Mahua Flower collected from different accession from BUAT Campus.

**Plant Name**	**Skin Strength(g)**	**Elasticity (mm)**	**Hardness**	**springiness**	**gumminess**	**Chewiness**	**Appearance**	**Juiciness**	**Texture**	**Aroma**	**Sweetness**
**BM-1**	112.91±22.86	1.918±0.1	96.78±0.12	95.61±0.68	44.03	41.8894	5.96±0.35	5.865±5.8	6.81±0.85	5.26±1.1	4.48±0.83
**BM-2**	99.526±10.24	2.636±0.42	98.268±.23	86.28±0.32	28.82±0.34	25.5958	5.75±3.3	6.48±3.75	5.83±0.44	6.09±0.27	5.92±0.25
**BM-3**	78.764±8.84	4.562±1.2	74.958±0.13	89.58±0.34	30.49±0.34	26.435	7.13±4.1	7.115	6.90±0.172	6.37±0.39	6.22±0.19
**BM-4**	85.634±15.43	3.18±0.6	91.3118±0.45	103.91±0.66	39.14±0.28	41.28±0.82	5.33±3.08	4.835	5.37±0.17	6.87±1.2	7.63±0.06
**BM-5**	67.678±13.50	3.232±0.77	71.3504±0.34	95.21±0.66	32.69±0.06	31.92±0.52	7.14±4.1	8.33	7.01±1.4	8.1±0.39	8.18±0.44
**BM-6**	139.142±37.65	3.088±0.74	136.5934±0.22	68±0.27	41.47±0.34	40.82±0.14	6.815±3.9	6.84	6.64±0.48	7.01±0.54	7.33±1.08
**BM-7**	78.61±9.23	5.95±0.98	86.7128±0.21	79.9±0.82	44.03±0.25	41.88±0.94	6.455±3.7	6.79	6.69±0.305	6.61±0.25	6.78±0.19
**BM-8**	62.52±10.32	4.81±0.54	92.3±0.25	76.23±0.89	28.82±0.34	25.59±0.58	7.49±4.3	7.415	7.22±0.14	6.98±0.16	6.89±0
**BM-9**	56.17±5.28	5.96±0.65	84.56±0.26	69.55±0.42	30.49±0.41	26.43±0.15	7.15±4.1	7.59	7.30±0.67	7.023±0.80	6.56±0

**BM-10**	130.904±30.45	4.938±0.83	115.1698±0.35	83.88±0.34	50.11±0.98	42.41±0.38	8.445±4.8	7.825	8.12±0.42	6.50±1.3	5.88±0.38
**BM-11**	76.64±7.45	5.61±0.43	94.689±0.31	93.56±0.43	39.14±0.28	41.28±0.82	6.175±3.5	5.525	5.71±0.43	5.75±0.48	5.92±0.25
**BM-12**	83.12±14.29	5.83±0.65	84.506±0.29	94.23±0.21	32.69±0.06	31.92±0.52	7.595±4.3	7.65	7.64±0.47	6.74±1.02	6.22±0.19
**BM-13**	90.59±16.24	5.72±0.74	66.456±0.24	68±0.23	41.47±0.34	40.82±0.14	8.34±4.8	7.745	7.72±0.98	7.08±0.90	6.56±0
